# External Validation of the Briganti Nomogram to Predict Lymph Node Invasion in Prostate Cancer—Setting a New Threshold Value

**DOI:** 10.3390/life11060479

**Published:** 2021-05-25

**Authors:** Bartosz Małkiewicz, Kuba Ptaszkowski, Klaudia Knecht, Adam Gurwin, Karol Wilk, Paweł Kiełb, Krzysztof Dudek, Romuald Zdrojowy

**Affiliations:** 1 Department of Urology and Oncologic Urology, Wroclaw Medical University, 50-556 Wroclaw, Poland; klaudia.knecht@gmail.com (K.K.); gurwin.adam@gmail.com (A.G.); karolwilk@me.com (K.W.); pk.kielb@gmail.com (P.K.); romuald.zdrojowy@umed.wroc.pl (R.Z.); 2 Department of Clinical Biomechanics and Physiotherapy in Motor System Disorders, Faculty of Health Science, Wroclaw Medical University, Grunwaldzka 2, 50-355 Wroclaw, Poland; kuba.ptaszkowski@umed.wroc.pl; 3 Faculty of Mechanical Engineering, Wroclaw University of Science and Technology, 50-370 Wrocław, Poland; krzysztof.dudek@pwr.edu.pl

**Keywords:** prostate cancer, radical prostatectomy, pelvic lymph node dissection, lymph node invasion, preoperative nomogram

## Abstract

(1) Introduction: The study aimed to test and validate the performance of the 2012 Briganti nomogram as a predictor for pelvic lymph node invasion (LNI) in men who underwent radical prostatectomy (RP) with extended pelvic lymph node dissection (PLND) to examine their performance and to analyse the therapeutic impact of using a different nomogram cut-off. (2) Material and Methods: The study group consisted of 222 men with clinically localized prostate cancer (PCa) who underwent RP with ePLND between 01/2012 and 10/2018. Measurements included: preoperative PSA, clinical stage (CS), primary and secondary biopsy Gleason pattern, and the percentage of positive cores. The area under the curve (AUC) of the receiver operator characteristic analysis was appointed to quantify the accuracy of the primary nomogram model to predict LNI. The extent of estimation associated with the use of this model was graphically depicted using calibration plots. (3) Results: The median number of removed lymph nodes was 16 (IQR 12–21). A total of 53 of 222 patients (23.9%) had LNI. Preoperative clinical and biopsy characteristics differed significantly (all *p* < 0.005) between men with and without LNI. A nomogram-derived cut-off of 7% could lead to a reduction of 43% (95/222) of lymph node dissection while omitting 19% (10/53) of patients with LNI. The sensitivity, specificity, and negative predictive value associated with the 7% cut-off were 81.1%, 50.3%, and 96.3%, respectively. (4) Conclusions: The analysed nomogram demonstrated high accuracy for LNI prediction. A nomogram-derived cut-off of 7% confirmed good performance characteristics within the first external validation cohort from Poland.

## 1. Introduction

In Europe, prostate cancer (PCa) is the most common cancer in men, accounting for 24% of all cancers diagnosed in 2018, equivalent to 450,000 new cases [1]. Poland ranks first in the incidence rates for men and second in the list of causes of cancer deaths (approx. 9.5%) [2]. Despite the widespread use of screening tests by determining PSA’s level, some patients are still diagnosed with a high local stage at diagnosis and are referred to as high risk on the D’Amico scale [3]. There is no doubt that radical treatment brings a much more significant benefit in overall survival and cancer-specific survival. Moreover, radical prostatectomy was most beneficial in patients with localised and locally advanced PCa [4,5]. Pelvic lymph node dissection (PLND) represents a vital staging procedure in identifying patients with lymph node invasion (LNI) and should be performed in patients with intermediate or high-risk PCa and omitting patients with the low-risk disease [6]. It allows selecting lymph nodes affected by the neoplastic invasion out of all the collected ones [7]. However, this procedure carries a risk of complications; therefore, it should be avoided if the risk of LNI is low. The decision to undertake a given treatment strategy depends on the preoperative PSA level, clinical stage, Gleason grade, histopathological examination and currently supported by new imaging techniques, in particular multiparametric MRI. Since the primary tumour is the source of growth factors most likely responsible for the localization of distant metastases, it should be treated as effectively as possible, while minimizing any complications.

Several studies have shown that the use of extended lymphadenectomy (ePLND) is recommended for each PLND indication [8,9,10]. To date, several predictive models have been developed to determine the risk of LNI in patients undergoing ePLND. The two most used (2021 Briganti and MSKCC) have been externally validated [11,12]. The developed predictive models require periodic checks to ensure their current patients’ accuracy. The result is a very accurate nomogram after internal validation. However, the lack of external validation is an obstacle to implementing the nomogram into broad clinical practice [13,14]. It is also impossible to obtain older patient data due to the different, more favourable grading of PCa in modern patients [15,16]. Finally, according to the European Association of Urology guidelines, ePLND should be performed for patients when the predicted probability of LNI exceeds 5% in Briganti calculation. However, in a few recent reports, 7% was suggested as an optimal cut-off with similar sensitivity and specificity, and a higher number of patients for whom PLND could be safely omitted [6,17]. Our study aimed to update and verify the nomogram predicting LNI on a different external patient data set and to find the most accurate cut-off for performing ePLND.

## 2. Materials and Methods

The data of 638 patients who underwent radical prostatectomy with ePLND due to a high-risk prostate cancer according to the d’Amico scale (PSA > 20 ng/mL, clinical stage ≥ T2c or biopsy Gleason sum 8–10) have been retrospectively studied. The collected data comes from 01/2012 to 10/2018 from the Clinical Department of Urology and Urology Oncology in Wrocław. Overall, 222 patients met the criteria—they had information on preoperative PSA, age, Gleason score, clinical stage, and had at least 8 fully described sections taken during ePLND.

The clinical stage of the tumour was assessed according to the updated TNM classification from 2016; the prostate biopsy was obtained by TRUS-guided systemic biopsy, and PSA was determined before the DRE examination [18]. Dedicated uropathologists performed the pathologic analysis of the biopsy and post-operative specimens following the International Society of Urological Pathology’s modifications in 2014 [19,20]. All specimens were collected and tested under the Stanford protocol guidelines, and their staging was determined according to the American Committee’s guidelines for the Staging System for Prostate Cancer [21,22]. Patients were preoperatively examined for metastases using abdominal CT with contrast and bone scintigraphy. An updated Briganti nomogram was calculated for each subject in this group based on age, PSA, TNM stage, Gleason score, and the percentage of samples taken [23].

Open radical prostatectomy was performed with the ascending technique, and in laparoscopic cases, transpertoneal access was used. The extent of the lymph node dissection was the same regardless of the surgical technique (open or laparoscopic). Extended pelvic lymphadenectomy (ePLND) involves removing fatty tissue from the obturator fossa area (along the obturator nerve and the external iliac vein) along the internal and external iliac arteries, extending to the distal segment of the common iliac artery. The lateral border is the pelvic wall, and the middle is the perivesical fat. The distal margin is the deep femoral vein. Each station is collected separately according to its anatomical location for selective histopathological examination [24].

This retrospective study was conducted in agreement with the Declaration of Helsinki of 1975, revised in 2013, and approved by the Ethics Committee of Wrocław Medical University (KB/545/2020).

## 3. Statistical Analysis

Descriptive statistics focus on the frequencies and proportions of categorical variables. Means, medians, and interquartile ranges are presented for continuously coded variables. The Chi-square and t-tests for the independent sample were used to compare the statistical significance of differences, respectively, of proportions and means. Analyses focused on testing the accuracy and calibration of a previously updated and internally validated nomogram to predict the likelihood of LNI in ePLND. Therefore, this nomogram was externally validated using predefined regression coefficients. The area under the curve (AUC) of the receiver operator characteristic analysis was used to quantify the model accuracy for LNI prediction. The extent of the overestimation or underestimation was investigated graphically in random calibration plots. Like Briganti, the specificity, sensitivity, and negative predictive value (NPV) were systematically assessed for each LNI probability threshold obtained from the nomogram [25].

All tests were two-sided with statistical significance set at *p* < 0.05. The analyses were performed using the statistical package for R (R base for statistical calculations, version 2.1.13).

## 4. Results

The characteristics of 222 patients and the primary cohort, consisting of the base for the nomogram, are presented in comparative Table 1. Additionally, the table’s data have been divided according to the occurrence of lymph node involvement (LNI) in the study group. Overall, LNI was found in 23.9% of patients (*n* = 53). The mean PSA value for patients with lymph node involvement was 24 ng/mL compared to 12.2 ng/mL without LNI, IQR: 12.7–33.8 vs. 7.2–17.6, respectively, with *p* < 0.001. Overall, patients with LNI had a higher clinical stage (T3) than those without, 41.5% vs. 13.1%, respectively (*p* < 0.001). Measurement of the biopsy secondary Gleason pattern also showed higher values in patients with LNI (52.8%) than without (21.9%, *p* < 0.001). The mean number of positive cores (6 vs. 5, *p* = 0.001), as well as the mean percentage of positive cores (50% vs. 42%, *p* < 0.001), were significantly higher in patients with LNI. The description of other pathological features is also listed in Table 1. 

**Figure 1:**
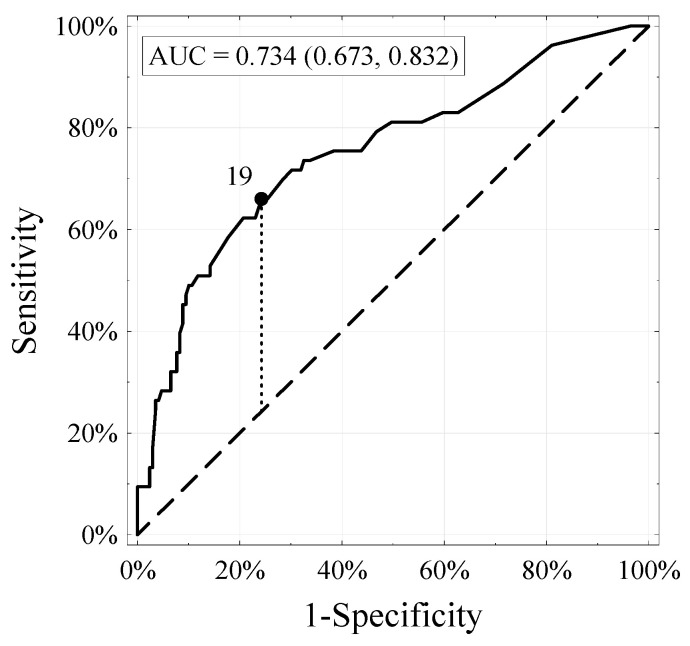
Receiver–operator characteristic (ROC) and area under the curve of the updated Briganti nomogram in 222 patients with risk of LNI.

The accuracy of the external validation performed was estimated at 0.734 (*n* = 222). Figure 1 shows the ROC calibration curve, demonstrating the dependence of specificity (X-axis) on sensitivity (Y-axis). A designated segment at an angle of 45° defines the ideal relationship between specificity and sensitivity for a given test. Points above this segment suggest that sensitivity is superior to specificity, which means that there are too many false positives versus false negatives. The opposite dependence occurs in the case of points located below this section. The entire calibration curve for our external validation of the nomogram runs above it, which means that at the moment, with the help of the nomogram, we are incorrectly finding too many false LNIs. However, the degree of over-detection is low due to the entire assay’s high accuracy.

Table 2 shows the probability of LNI occurrence resulting from applying the Briganti nomogram in the cohort where external validation was performed. For each cut-off point of the nomogram, the actual number of men with and without LNI was calculated. In addition, the sensitivity, specificity, positive predictive value (PPV), and negative predictive value (NPV) for the individual cut-off values of the nomogram were characterized. ePLND could be omitted in 95 men (42.8%), but this group would include 10 patients with LNI (18.9% of all LNI patients) using the nomogram cut-off of 7%. The sensitivity and specificity of the 7% cut-off were 81.1% and 50.3%, respectively, and NPV and PPV were 96.3% and 33.9%, respectively.

## 5. Discussion

According to the latest EAU guidelines, the ePLND template is recommended whenever PLND is required [8,9,10,26]. During ePLND, at least 13 lymph nodes should be removed and investigated to achieve optimal staging accuracy. In cases with 13 or more lymph nodes examined, the rate of metastatic involvement is twice as high as in lower lymph node counts [27]. Moreover, it has been proven that the more lymph nodes are removed, the more accurate the staging will be [8,28]. In our study, the median value of removed lymph nodes was 16, which allowed for an accurate assessment. There are different LNI predictive nomograms [11,29,30,31,32]. Our research performed an external validation of the Briganti nomogram for the Polish cohort [23]. Thus far, it has not been checked and formalized for the Polish centre’s needs. Our main goal was to optimize the local cohort nomogram in patients after radical prostatectomy. We tested different cut-off values that could be used to define with the highest accuracy patients in whom ePLND should be executed.

It is important to avoid unnecessary lymphadenectomy due to its intra- and postoperative complications. ePLND extends surgery time by an average of 90 min, which increases blood loss and the risk of ischemic complications [28,33]. It can also cause obturator nerve injury, life–threatening bleeding due to iliac vessels laceration, ureteral injury, deep venous thrombosis, pulmonary embolism, and lymphocele [34,35]. The latest reports indicate the need to change the cut-off value for performing ePLND at RP from 5% to 7%, resulting from the nomogram [17]. Using a 7% nomogram cut-off in Diamand et al.’s study allows the avoidance of 55.9% of PLNDs, while omitting less than 2.6% of patients with LNI [36]. Venclovas et al.’s nomogram-derived cut-off of 7% is associated with a risk of missing LNI in 4%, avoiding unnecessary surgeries in 47% [17]. However, Hansen et al. decide to use a 4% cut-off to reduce 48% of lymph node dissection, while omitting 10% of patients with LNI [37].

Performed analyses showed some critical findings. Firstly, patients undergoing ePLND in different clinical centres may show very different clinical stages and pathological neoplastic changes. Two components are particularly noticeable compared to the primary medium where the Briganti nomogram was developed [23]. In our clinic, the frequency of LNI 23.9% compared to only 8.3% in the original series shows that some centres operate on patients at a higher stage of advancement than others. This fact may significantly affect the effectiveness of the prediction tools used, as in some centres, less aggressive tumours are removed. Secondly, we recorded a higher degree of malignancy in the Gleason primary and secondary patterns than in Briganti’s group. In conclusion, our data clearly show that similar cohorts of men with prostate cancer may differ in terms of tumour characteristics, which means that external, cohort–specific validation is required before using a prognostic tool in routine clinical practice.

After testing as part of our external validation on an independent cohort, the nomogram’s predicted accuracy was 73.4%, preferably compared to the 87.6% obtained by Briganti’s internal validation team. The similar overall accuracy of the internal and external validation results indicates that, despite significant discrepancies in biopsy advancement and LNI operations frequency, this nomogram can adjust to these differences with a slight loss of accuracy. It follows that the nomogram’s overall accuracy can be expected to remain similar, even if the target population differs from the original cohort. However, differences indicate that the initially optimal cut-off value will not be ideal for other cohorts.

We analysed many different potential cut-off values, comparing them with the results obtained by Briganti’s team, to determine the best one for our cohort. In the original series, a threshold of 5% was adopted. In the studied group, the value that separates patients in whom ePLND should be performed from patients in whom ePLND should be omitted is 7%. This value is the optimal compromise between the number of avoided ePLNDs (42.8% of all patients) compared to the number of missed LNI patients. (18.9% of all LNI patients) [38]. Alternatively, using the proposed initial 5% cut-off, we would have to perform ePLND on a much larger number of men (66.3% vs. 57.2%), and only a small number of patients with LNI would benefit from it (false negative 17% vs. 19%). Despite our choice of a cut-off value of 7%, different sites may choose a different cut-off point that is optimal for their cohort. If the acceptable compromise between the number of ePLNDs performed and the missed LNIs is considered too high, a lower cut-off should be chosen. Conversely, a higher cut-off value may be considered when dealing with a population of patients with better prognostic characteristics and a less malignant course.

The study’s overall accuracy is one of the few critical benchmarks in the predictive tool. Calibration or correlation between predicted and observed indicators represents another key volatility. In particular, the first one shows the operation of the prognostic tool for a specific risk group in the studied population. In the key range of values, it can assess, in detail, the relationship between the observed LNI risk and the predicted one using the nomogram. This range is 0–10%, and within its range, there should be a cut-off point at which ePLND will not be performed. More than 10% of specialists, based on the patient’s clinical picture, would be inclined to perform this procedure. Therefore, the nomogram’s proper calibration is the most essential for this key cut-off range. It includes the grey area of the uncertainty of the need to perform the ePLND. It is noteworthy that the nomogram’s calibration was not perfect and revealed an overestimation in terms of the predicted LNI probability. It was insignificant, which indicates the predictive stability of LNI occurrence using this nomogram. This discovery requires meticulous consideration, indicating the appropriate cut-off value. Therefore, it is essential to remember and carefully analyse the potential source of a possible error and be cautious when making final clinical decisions.

Despite its value, our study is not without limitations. First of all, the population compared to external validation in this study was smaller than in the development cohort of the updated LNI nomogram, which includes patients admitted to one Polish tertiary centre. As discussed earlier, validations from numerous institutions, preferably international, could lead to obtain more generalized conclusions. The previous analysis of the multi–institutional cohort, showed significant differences in accuracy between the various external validations [39]. Nevertheless, there may be problems with the data from many institutions, especially in predicting LNI, before lymphadenectomy. It is important to mention that, despite the known perception of performing ePLND instead of PLND, the standards or scope of this procedure can be different [10].

Furthermore, due to the scientific development on PLND over the years, the calendar year of the operation performed may affect the number of lymph nodes collected [40]. Surgical methods can also vary (open prostatectomy vs. laparoscopic prostatectomy), which is relevant for drawing conclusions [41,42]. Even though every surgeon decided on the same ePLND scope, differences in lymph node detection can still be noticed as a result of various operation methods or specialist’s experience [43].

There may also be differences with the templates that were used in ePLND. Mattei and colleagues carefully checked the prostate’s primary lymphatic landing site, founding that only 63% of the lymph nodes will be removed during classical ePLND [44]. In addition to this extent, a resection of the lymph nodes alongside the common iliac arteries to the crossing of the ureter could improve the percentage to 75%. Consequently, another external validation may result in different estimated accuracy. Moreover, patients were somehow pre-selected for ePLND before RP due to the previous nomogram. Despite this fact, the updated nomogram can still be verified in the current patient cohort. Lastly, our study’s retrospective character is another limitation that may have impacted the results.

## 6. Conclusions

In conclusion, the external validation of the Briganti nomogram on the Polish cohort shows good accuracy and precise calibration. The cut-off value of the data calculated by the nomogram was optimized to 7%, giving better results than the proposed threshold of 5%. Additional external validation studies should be performed, and the predictive value adjusted to the local cohort.

## Figures and Tables

**Table 1:** Clinical and pathological data of primary and current study cohorts [25].

	Comparison between Primary and Current Study Cohorts:			Comparison within Study Cohort:		
	Primary (2006–2010) [25]	Current (2012–2018)	*p*	LNI (−)	LNI (+)	*p*
No (%)	588 (–)	222 (–)		169 (76.1)	53 (23.9)	
Age, years						
Median	66	65	<0.001	64	66	0.045
IQR	60–70	60–68		59–68	62–70	
PSA, ng/mL						
Median	6.3	13.6	<0.001	12.2	24.0	<0.001
IQR	4.8–8.9	7.6–21.1		7.2–17.6	12.7–33.8	
No. of biopsy cores taken						
Median	17	12	<0.001	12	12	0.639
IQR	13–24	12–12		12–12	10–12	
No. of positive biopsy cores						
Median	6	5	<0.001	5	6	0.001
IQR	3–10	3–8		3–7	4–10	
Perc. of positive biopsy cores						
Median	36	42	0.296	42	50	<0.001
IQR	17–61	25–66		25–58	33–91	
Clinical stage:						
T1	373 (63.4)	10 (4.5)	<0.001	8 (4.7)	2 (3.8)	<0.001
T2	184 (31.3)	168 (75.7)		139 (82.2)	29 (54.7)	
T3	31 (5.3)	44 (19.8)		22 (13.1)	22 (41.5)	
Primary biopsy Gleason pattern:						
≤3	488 (83.0)	155 (69.8)	<0.001	130 (76.9)	25 (47.2)	<0.001
≥4	100 (17.0)	67 (30.2)		39 (23.1)	28 (52.8)	
Secondary biopsy Gleason pattern:						
≤3	406 (69.0)	157 (70.7)	0.707	132 (78.1)	25 (47.2)	<0.001
≥4	182 (31.0)	65 (29.3)		37 (21.9)	28 (52.8)	
Clinical risk classification:						
Low		16 (7.8)		15 (9.6)	1 (2.0)	<0.001
Intermediate		45 (22.0)		44 (28.2)	1 (2.0)	
High		144 (70.2)		97 (62.2)	47 (96.0)	
Pathological stage:						
T2	431 (73.3)	108 (48.6)	<0.001	103 (60.9)	5 (9.4)	<0.001
T3a	97 (16.5)	48 (21.6)		33 (19.5)	15 (28.3)	
T3b	58 (9.9)	66 (29.7)		33 (19.5)	33 (62.3)	
T4	2 (0.3)	0 (0.0)		0 (0.0)	0 (0.0)	
Pathological primary Gleason pattern:						
≤3		141 (63.5)		119 (70.4)	25 (47.2)	0.003
≥4		81 (36.5)		50 (29.6)	28 (52.8)	
Pathological secondary Gleason pattern:						
≤3		142 (64.0)		119 (70.4)	23 (43.4)	<0.001
≥4		80 (36.0)		50 (29.6)	30 (56.6)	
Number of positive lymph nodes						
Median	2	2	<0.001	0	2	<0.001
IQR	1–3	1–5		0–0	1–5
Number of lymph nodes removed						
Median	19	16	<0.001	15	20	<0.001
IQR	15–25	12–21		10–20	16–26	
Biopsy Gleason Grading Group						
1		76 (34.2)		64 (37.9)	12 (22.7)	<0.001
2		52 (23.4)		46 (27.2)	6 (11.3)	
3		29 (13.1)		22 (13.0)	7 (13.2)	
4–5		65 (29.3)		37 (21.9)	28 (52.8)	
Pathological Gleason Grading Group						
1		26 (11.7)		26 (15.4)	0 (0.0)	<0.001
2		58 (26.1)		49 (29.0)	9 (17.0)	
3		58 (26.1)		44 (26.0)	14 (26.4)	
4–5		80 (36.1)		50 (29.6)	30 (56.6)	

*n* (%) or median [IQR], IQR: interquartile range, LNI: lymph node invasion, PSA: prostate-specific antigen.

**Table 2:** Analyses of the Nomogram-Derived Cut-Offs of the Externally Validated Updated LNI Nomogram.

Cut-off, %	TN + FN	TN	FN	TP + FP	FP	TP	NPV	PPV	TPR	TNR
1	6 (3.6)	6 (3.6)	0 (0)	216 (97.3)	163 (96.4)	53 (100)	100	24.5	100	3.6
2	34 (15.3)	32 (18.9)	2 (3.8)	188 (84.7)	137 (81.1)	51 (96.2)	94.1	27.1	96.2	18.9
3	54 (24.3)	48 (28.4)	6 (11.3)	168 (75.7)	121 (71.6)	47 (88.7)	88.9	28.0	88.7	28.4
4	73 (32.9)	64 (37.9)	9 (17.0)	150 (67.6)	106 (62.7)	44 (83.0)	97.4	29.3	83.0	37.6
5	77 (34.7)	68 (40.2)	9 (17.0)	145 (65.3)	101 (59.8)	44 (83.0)	97.2	30.3	83.0	40.2
6	85 (38.3)	75 (44.4)	10 (18.9)	137 (61.7)	94 (55.6)	43 (81.1)	96.9	31.4	81.1	44.4
7	95 (42.8)	85 (50.3)	10 (18.9)	127 (57.2)	84 (49.7)	43 (81.1)	96.3	33.9	81.1	50.3
8	101 (45.5)	90 (53.3)	11 (20.8)	121 (54.5)	79 (46.7)	42 (79.2)	95.6	34.7	79.2	53.3
9	108 (48.6)	95 (56.2)	13 (24.5)	114 (51.4)	74 (43.8)	40 (75.5)	96.3	35.1	75.5	56.2
10	112 (50.5)	99 (58.6)	13 (24.5)	110 (49.5)	70 (41.4)	40 (75.5)	95.0	36.4	75.5	58.6
15	133 (59.9)	118 (69.8)	15 (28.3)	89 (40.1)	51 (30.2)	38 (71.7)	93.7	42.7	71.7	69.8
20	154 (69.4)	134 (79.3)	20 (37.7)	68 (30.6)	35 (20.7)	33 (62.3)	93.3	48.5	62.3	79.3
25	170 (76.6)	145 (85.8)	25 (47.2)	51 (23.0)	24 (14.2)	27 (50.9)	92.5	52.9	51.9	85.8
30	179 (80.6)	152 (89.9)	27 (50.9)	43 (19.4)	17 (10.1)	26 (49.1)	91.6	60.5	49.1	89.9

Exemplary cutoffs with calculated ability to identify patients with (*n* = 53) or without (*n* = 169) pathologically confirmed LNI. TN + FN: patients below recommended ePLND cut-off, TN: patients below cut-off without pathologic LNI, FN: patients below cut-off with pathologic LNI, TP + FP: patients above recommended ePLND cut-off, FP: patients above cut-off without pathologic LNI, TP: patients above cut-off with pathologic LNI, NPV: negative predictive value, PPV: positive predictive value, TPR: sensitivity, TNR: specificity.

## Data Availability

The datasets analysed are available from the corresponding author on reasonable request.
